# Trousseau Syndrome Associated With Prostate Cancer: A Systematic Review of Case Reports

**DOI:** 10.7759/cureus.105922

**Published:** 2026-03-26

**Authors:** Shiho Amano, Ryuichi Ohta

**Affiliations:** 1 Community Care, Unnan City Hospital, Unnan, JPN

**Keywords:** arterial thrombosis, cancer-associated thrombosis, hypercoagulability, prostate cancer, systematic review, trousseau syndrome, venous thromboembolism

## Abstract

Trousseau syndrome is a well-recognized manifestation of cancer-associated hypercoagulability, characterized by recurrent or migratory thrombotic events. Although thrombotic complications have been reported in various malignancies, the clinical characteristics of Trousseau syndrome associated with prostate cancer remain poorly described. This study aimed to systematically review reported cases of prostate cancer-associated Trousseau syndrome and summarize their clinical features. A systematic literature search was conducted in PubMed, Embase, and Web of Science to identify relevant reports describing thrombotic events associated with prostate cancer. Full-text case reports and case series providing sufficient clinical details were included. Six cases met the inclusion criteria. The patients’ ages ranged from 55 to 87 years. Hypertension and dyslipidemia were the most commonly reported comorbidities. In half of the cases, thrombotic events preceded the diagnosis of prostate cancer. All cases with available staging information had stage IV disease, and reported Gleason scores ranged from 7 to 9. Thrombotic events occurred in diverse vascular territories and involved both the arterial and venous systems, including the carotid arteries, deep veins of the lower extremities, the thoracic aorta, retinal veins, and the internal jugular vein. Anticoagulation therapy, particularly intravenous heparin, was the most commonly reported treatment, although clinical outcomes varied among cases. These findings suggest that Trousseau syndrome associated with prostate cancer tends to occur in advanced disease and may occasionally represent the initial manifestation of malignancy; however, interpretation is limited by the small sample size and heterogeneity of reporting inherent to case-report-based evidence. Clinicians should consider underlying prostate cancer in patients presenting with unexplained thrombotic events, particularly in older men. Further studies are required to clarify the optimal management and prognostic implications of cancer-associated thrombosis in prostate cancer.

## Introduction and background

Trousseau syndrome is a clinical condition characterized by thrombotic events associated with malignancy-related hypercoagulability, resulting in arterial or venous thrombosis [1]. Originally described as migratory thrombophlebitis occurring in patients with cancer, the concept has since expanded to encompass a broader spectrum of recurrent, multiple, and refractory thrombotic events associated with malignancy. Trousseau syndrome has been particularly associated with mucin-producing adenocarcinomas, including pancreatic, gastric, lung, and ovarian cancers [2]. The underlying mechanisms are thought to involve activation of the coagulation cascade by tumor-derived factors, including tissue factor (TF), mucins, and inflammatory cytokines, which collectively promote a systemic hypercoagulable state [3].

Prostate cancer is one of the most common malignancies among men worldwide and remains a major contributor to cancer-related morbidity and mortality. Previous studies have shown that patients with prostate cancer, particularly those with advanced disease, have a significantly higher risk of venous thromboembolism (VTE), including deep vein thrombosis and pulmonary embolism (PE), compared with the general population [4]. This increased thrombotic risk is considered multifactorial, involving tumor-related activation of the coagulation system, reduced mobility or physical inactivity, and treatment-related factors such as androgen deprivation therapy (ADT) [5].

However, most thrombotic events reported in prostate cancer are related to the venous system. In contrast, cases presenting with arterial thrombosis or multiple systemic thrombotic events consistent with Trousseau syndrome are relatively rare. To date, reports of prostate cancer-associated Trousseau syndrome have largely been limited to isolated case reports or small case series, and no comprehensive review has systematically summarized their clinical characteristics, risk factors, pathophysiology, or outcomes [6,7].

In some patients with prostate cancer, thrombotic events may occur as the initial manifestation of the malignancy, whereas in others, they develop during the course of advanced disease and may involve both arterial and venous systems. In such situations, malignancy-associated hypercoagulability should be considered as a potential underlying mechanism.

Therefore, systematically collecting and analyzing reported cases of Trousseau syndrome in patients with prostate cancer may provide important insights into the timing of onset, tumor activity, treatment status, predominant sites of thrombosis, diagnostic approaches, and subsequent clinical outcomes. Such information could contribute to improved risk recognition, earlier diagnosis, and optimized management strategies for thrombotic complications in patients with prostate cancer.

The aim of this study was to systematically review reported cases of Trousseau syndrome associated with prostate cancer and to characterize the clinical features, including patient background, tumor stage, thrombosis sites, diagnostic methods, treatments, and outcomes. By synthesizing these findings, we sought to provide clinically relevant insights to support the diagnosis and management of this rare but important complication in prostate cancer.

## Review

Methods

Literature Search Strategy

To identify reported cases of Trousseau syndrome associated with prostate cancer, a literature search was conducted using three electronic databases: PubMed, Embase, and Web of Science, based on the Preferred Reporting Items for Systematic reviews and Meta-Analyses (PRISMA) guidelines [8]. Considering the substantial progress in cancer diagnosis and treatment in recent decades, the search was limited to studies published between January 2000 and April 2025. Search terms were developed by combining Medical Subject Headings (MeSH) terms with relevant free-text terms related to prostate cancer and malignancy-associated thrombosis. The full database-specific search strategies for PubMed, Embase, and Web of Science are provided in Appendix A. The primary search strategy included the following terms: ("Prostate cancer" OR "Prostatic neoplasms" OR "Prostate carcinoma") AND ("Trousseau syndrome" OR "Cancer-associated thrombosis" OR "Arterial thrombosis" OR "Ischemic stroke" OR "Hypercoagulability"). To increase the sensitivity of the search, variations of these keywords were also included in title and abstract searches. Only articles published in English were included to ensure consistency in data extraction and interpretation. Conference abstracts, unpublished studies, and gray literature were excluded because they had not undergone formal peer review.

Study Selection

Studies reporting clinical cases of prostate cancer associated with malignancy-related thrombosis consistent with Trousseau syndrome were considered eligible for inclusion. Eligible reports included case reports and case series describing individual patient-level clinical information. Studies were excluded if the diagnosis of Trousseau syndrome was unclear, if thrombosis was clearly attributable to malignancies other than prostate cancer, or if individual clinical data could not be extracted. Basic science studies, animal experiments, review articles, editorials, letters, and abstract-only publications were also excluded.

Data Extraction and Synthesis

Relevant clinical information was extracted from each eligible study and summarized descriptively. Extracted variables included patient demographics (age, sex, and race when available), prostate cancer characteristics (tumor stage, Gleason score, prostate-specific antigen (PSA) level, and treatment history), and details of thrombotic events. Information related to thrombosis included the timing of thrombosis relative to prostate cancer diagnosis, anatomical location of thrombosis, diagnostic methods (e.g., MRI, CT, ultrasonography, or laboratory testing), and therapeutic interventions such as anticoagulation or thrombolytic therapy. Clinical outcomes, including recovery, recurrence, residual deficits, and mortality, were also recorded when available. Given the limited number of reported cases and the heterogeneity of available data, findings were synthesized descriptively. Continuous variables were summarized using reported ranges or averages when available, and categorical variables were summarized as frequencies.

Risk of Bias Assessment

A risk of bias assessment was conducted for all included studies using the Joanna Briggs Institute (JBI) Critical Appraisal Checklist for Case Reports [9]. This tool evaluates methodological quality across eight domains: patient demographic characteristics, patient history, clinical condition, diagnostic assessment, intervention details, post-intervention outcomes, adverse events, and clarity of case reporting. Each item was assessed as “Yes,” “No,” “Unclear,” or “Not applicable.” Two reviewers independently evaluated the included studies, and disagreements were resolved through discussion. Given that all included studies were case reports, the assessment was intended to evaluate reporting quality and potential sources of bias rather than to exclude studies from the analysis.

Results

Study Selection

**Figure 1:**
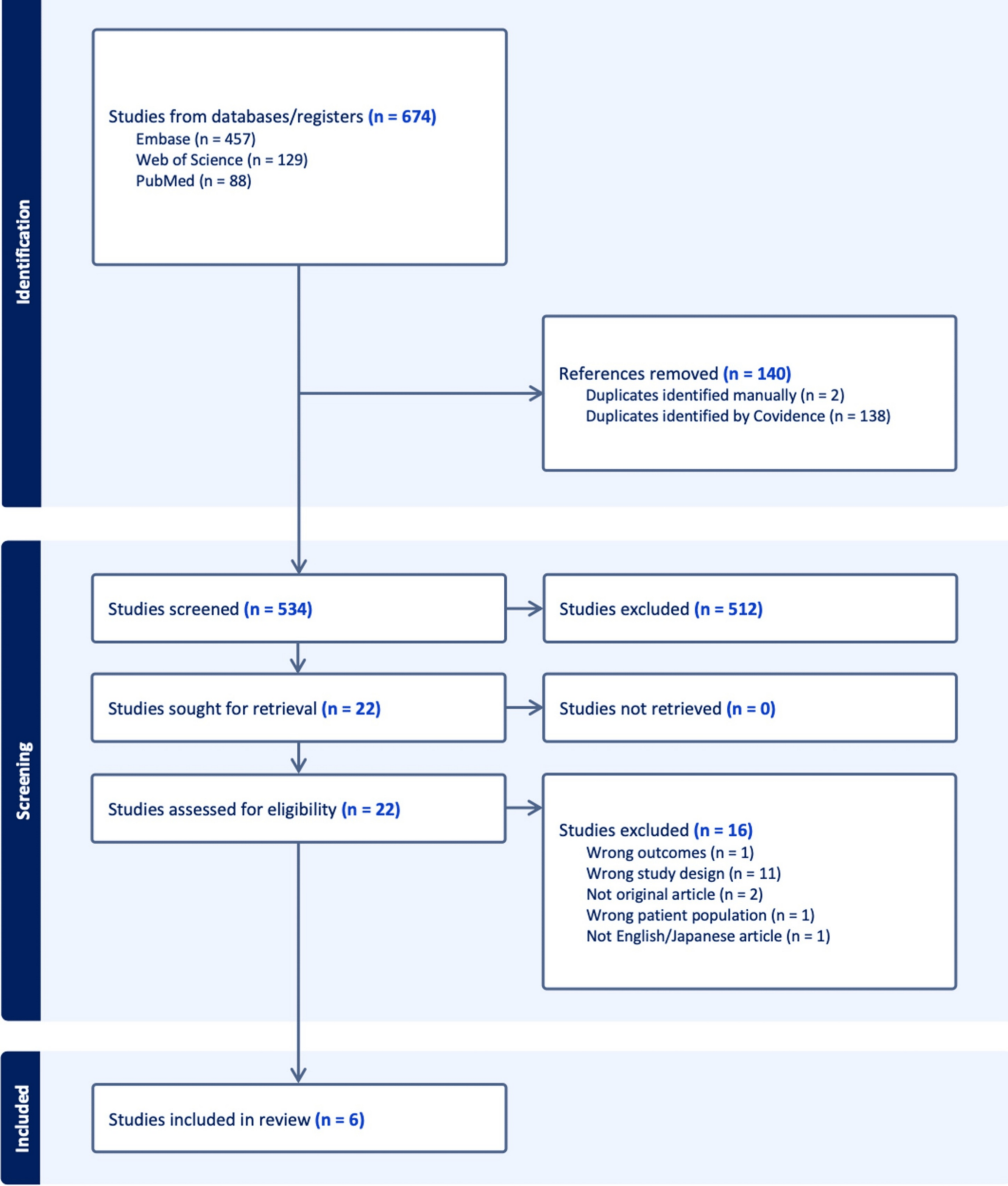
Flow diagram of the study selection

The literature search identified a total of 674 records from three databases: Embase (n = 457), Web of Science (n = 129), and PubMed (n = 88). After removing duplicate records, 534 articles remained for title and abstract screening. Of these, 512 articles were excluded because they did not meet the eligibility criteria. The remaining 22 articles underwent full-text review. Among them, 16 articles were excluded for the following reasons: inappropriate outcomes (n = 1), incompatible study design (n = 11), non-original article (n = 2), ineligible study population (n = 1), and publication in languages other than English or Japanese (n = 1). Ultimately, six case reports met the inclusion criteria and were included in the final analysis (Figure 1).

Patient Characteristics

A total of six patients with prostate cancer-associated Trousseau syndrome were identified. The patients ranged in age from 55 to 87 years, with a mean age of 70.0 years. Ethnicity was reported in three cases (50%), including Sri Lankan, Chinese, and Caucasian, whereas the remaining three cases (50%) did not report ethnicity. Most patients had underlying comorbidities. Hypertension was the most frequently reported comorbidity, present in four patients (67%), followed by dyslipidemia in three patients (50%). Other reported comorbidities included diabetes mellitus, coronary artery disease, gout, gastric ulcer, chronic lung disease, intracranial hemorrhage, and glaucoma. One patient (17%) had no reported comorbidities. Regarding the timing of thrombotic events relative to the diagnosis of prostate cancer, three patients (50%) developed thrombosis before the diagnosis of prostate cancer, whereas three patients (50%) developed thrombosis after the diagnosis of prostate cancer. Among these, one patient developed thrombosis two months after biochemical recurrence of prostate cancer. Tumor stage was reported in five cases, and all five patients (100%) had stage IV disease. Gleason score was reported in four cases and ranged from 7 to 9, indicating intermediate- to high-grade tumors. PSA levels were available in four cases and ranged from 125.2 to 2,464 ng/mL (Table 1).

**Table 1:** Clinical characteristics of reported cases of prostate cancer-associated Trousseau syndrome This table summarizes the demographic and clinical characteristics of the six reported cases included in this review. Information includes patient age, ethnicity, comorbidities, timing of prostate cancer diagnosis relative to thrombotic events, tumor stage, and Gleason score. The timing of diagnosis indicates whether prostate cancer was diagnosed before or after the thrombotic presentation. PSA, prostate-specific antigen

Author	Age (years)	Ethnicity	Past medical history	Time of diagnosis	Stage	Gleason score	PSA (ng/mL)
Rodriguez and Walsh (2000) [4]	55	Not stated	None	One year before thrombosis presentation	Stage IV	4 + 3 = 7	125.2
Bandara et al. (2016) [5]	75	Sri Lankan	Hypertension, hyperlipidemia, intracranial hemorrhage	After thrombosis presentation	Stage IV	3 + 4 = 7	257
Castro-Navarro et al. (2015) [9]	72	Not stated	Hypertension, hyperlipidemia, gout, coronary artery disease, primary open-angle glaucoma (bilateral), chronic lung disease	Before thrombosis presentation; biochemical recurrence found two months prior to thrombosis presentation	Not stated	Not stated	Not stated
Kwong et al. (2017) [10]	64	Chinese	Hypertension, hyperlipidemia, diabetes mellitus	Six years before thrombosis presentation	Stage IV	4 + 5 = 9	272
Kohada et al. (2019) [11]	67	Not stated	Gastric ulcer	After thrombosis presentation	Stage IV	5 + 4 = 9	2,464
Wagner and Lantz (2024) [12]	87	Caucasian	Hypertension, chronic lung disease	After thrombosis presentation	Stage IV	Not stated	Not stated

Characteristics of Thrombotic Events

The anatomical locations of thrombosis varied widely and involved both the arterial and venous systems. Reported thrombotic sites included the internal jugular vein and brachiocephalic vein, cerebral arteries, branch retinal veins, the thoracic aorta, and the deep veins of the lower extremities. Among the reported cases, venous thrombosis was observed in three patients and arterial thrombosis in three patients. The most frequently reported clinical manifestation was cerebral infarction, which occurred in two cases. Various imaging modalities were used to confirm the diagnosis of thrombosis, depending on the affected organ. Diagnostic methods included CT, MRI, duplex ultrasonography, and ophthalmologic examinations such as fluorescein angiography and optical coherence tomography. These findings indicate that thrombotic events associated with prostate cancer may present in diverse vascular territories, reflecting the systemic hypercoagulable state characteristic of Trousseau syndrome (Table 2).

**Table 2:** Characteristics of thrombotic events in the included cases This table summarizes the characteristics of thrombotic events reported in the included case reports of prostate cancer-associated Trousseau syndrome. The table presents the type of thrombosis (arterial or venous), the anatomical site, and the diagnostic modalities used to confirm the thrombotic events in each case.

Author	Type of thrombosis	Site of thrombosis	Diagnostic modality
Rodriguez and Walsh (2000) [4]	Arterial	Branch retinal vein	Fundus examination and fluorescein angiography
Bandara et al. (2016) [5]	Arterial	Cerebral arteries (multiple cerebral infarctions)	Brain MRI
Castro-Navarro et al. (2015) [9]	Venous	Internal jugular vein and brachiocephalic vein	CT and ultrasonography
Kwong et al. (2017) [10]	Arterial	Thoracic aorta	CT
Kohada et al. (2019) [11]	Venous	Deep veins of the lower extremities	Duplex ultrasonography
Wagner and Lantz (2024) [12]	Venous	Deep veins of the lower extremities	Ultrasonography

Treatment and Clinical Outcomes

Anticoagulation therapy was the main treatment approach for thrombotic events associated with prostate cancer. Intravenous heparin was administered in four of the six cases (67%), whereas two patients (33%) did not receive anticoagulation therapy. Thrombolytic therapy was not performed in any of the reported cases. Regarding clinical outcomes, three patients (50%) showed improvement in thrombotic symptoms, including one patient who experienced partial improvement. One patient (17%) showed no clinical improvement, while two cases (33%) did not report detailed clinical outcomes. Recurrence of thrombosis was reported in one patient (17%), whereas three cases (50%) explicitly reported no recurrence, and two cases (33%) did not provide information regarding recurrence. Information regarding long-term survival was limited. One patient was reported to have died three years after the initial thrombotic event, while survival outcomes were not reported in the remaining cases (Table 3).

**Table 3:** Treatment and clinical outcomes in the included cases This table summarizes the therapeutic approaches and clinical outcomes of thrombotic events reported in the included case reports of prostate cancer-associated Trousseau syndrome. The table presents the use of anticoagulation therapy, thrombolytic therapy, recovery status of thrombosis, recurrence of thrombotic events, and reported survival duration after diagnosis.

Author	Anticoagulation	Thrombolysis	Thrombosis recovery	Recurrence	Survival duration
Rodriguez and Walsh (2000) [4]	Intravenous heparin	Not performed	Improved	Recurrence	Died three years after initial diagnosis
Bandara et al. (2016) [5]	Intravenous heparin	Not performed	Improved	No recurrence	Not stated
Castro-Navarro et al. (2015) [9]	Not performed	Not performed	Partial improvement	Not stated	Not stated
Kwong et al. (2017) [10]	Not performed	Not performed	No improvement	Not stated	Not stated
Kohada et al. (2019) [11]	Intravenous heparin	Not performed	Not stated	No recurrence	Not stated
Wagner and Lantz (2024) [12]	Intravenous heparin	Not performed	Not stated	No recurrence	Not stated

Risk of Bias Assessment

The risk of bias assessment using the JBI Critical Appraisal Checklist for Case Reports indicated that most included studies adequately reported patient demographic characteristics, clinical presentation, diagnostic findings, and key clinical lessons. However, several reports lacked detailed information on post-intervention follow-up and treatment-related adverse or unanticipated events. In addition, some studies provided limited detail regarding therapeutic interventions or longitudinal clinical course. Overall, the reporting quality of the included case reports was considered moderate, reflecting the inherent limitations of case-based evidence (Table 4).

**Table 4:** JBI Critical Appraisal Checklist for included case reports ^*^ Overall appraisal = number of checklist items rated “Yes” out of eight domains. This summary reflects reporting completeness and is not a validated quantitative risk of bias score. JBI, Joanna Briggs Institute

Study	Demographic characteristics	History and timeline	Clinical condition on presentation	Diagnostic assessment	Intervention details	Post-intervention condition	Adverse/unanticipated events	Takeaway lessons	Overall appraisal^*^
Rodriguez and Walsh (2000) [4]	Yes	Yes	Yes	Yes	Yes	Yes	Unclear	Yes	7/8
Bandara et al. (2016) [5]	Yes	Yes	Yes	Yes	Yes	Yes	Unclear	Yes	7/8
Castro-Navarro et al. (2015) [9]	Yes	Yes	Yes	Yes	Unclear	Yes	Unclear	Yes	6/8
Kwong et al. (2017) [10]	Yes	Yes	Yes	Yes	Unclear	Yes	Unclear	Yes	6/8
Kohada et al. (2019) [11]	Yes	Yes	Yes	Yes	Yes	Unclear	Unclear	Yes	6/8
Wagner and Lantz (2024) [12]	Yes	Yes	Yes	Yes	Yes	Unclear	Unclear	Yes	6/8

Discussion

Summary of the Study

This systematic review summarized the clinical characteristics of Trousseau syndrome associated with prostate cancer, focusing on the timing of thrombotic events, thrombotic sites, treatment strategies, and clinical outcomes. Trousseau syndrome was originally described as migratory thrombophlebitis associated with malignancy, but the concept has since expanded to include recurrent, multifocal, and treatment-resistant thrombotic events related to cancer [13,14]. These manifestations encompass not only VTE and arterial thromboembolism but also a wide spectrum of coagulation disorders, including disseminated intravascular coagulation, thrombotic microangiopathy, veno-occlusive disease, and nonbacterial thrombotic endocarditis (NBTE) [14]. Cancer-associated thrombosis is thought to arise from multiple mechanisms. Tumor cells can activate the coagulation cascade by releasing TF, inflammatory cytokines, and mucins, thereby promoting systemic hypercoagulability [15,16]. In addition, clinical factors such as tumor burden, infection, dehydration, immobilization, and the presence of central venous catheters may further enhance thrombotic risk. In the present review, thrombotic events occurred in both arterial and venous systems and involved various vascular territories, ranging from large vessels to microvascular structures. These findings are consistent with the systemic prothrombotic state characteristic of cancer-associated thrombosis.

Comparison With Previous Studies

The risk of thrombosis varies considerably depending on cancer type. Previous studies have shown that VTE occurs most frequently in pancreatic cancer, Hodgkin lymphoma, non-Hodgkin lymphoma, and ovarian cancer [17,18]. In contrast, arterial thrombotic events are more commonly reported in lung and colorectal cancers [19]. For prostate cancer, population-based studies have demonstrated that the risk of VTE is significantly increased after diagnosis. Compared with matched controls from the general population, patients with prostate cancer have an approximately threefold increased risk of VTE within the first 12 months after diagnosis (adjusted HR 3.0, 95% CI 2.7-3.3) [17]. Nevertheless, prostate cancer is generally not considered among the malignancies with the highest thrombotic risk. One prostate cancer-specific factor associated with thrombosis is ADT, which has been reported to increase the risk of thrombotic events [20].

Advanced disease stage is another important determinant of cancer-associated thrombosis. Several studies have shown that metastatic disease and advanced tumor stage are strongly associated with an increased risk of both venous and arterial thrombosis [19-21]. Similar findings have been reported in prostate cancer [22]. In the present review, all cases with available staging information had stage IV disease, and the reported PSA levels were markedly elevated, suggesting a large tumor burden. These observations support the hypothesis that advanced disease and increased tumor burden may contribute to the hypercoagulable state in prostate cancer. However, the limited number of cases warrants cautious interpretation.

Another notable finding of this review is that approximately half of the thrombotic events occurred before the diagnosis of prostate cancer. Previous studies have demonstrated that the risk of thrombosis begins to increase approximately 150 days before the diagnosis of cancer and peaks about 30 days before diagnosis [19]. The risk remains elevated during the first year after cancer diagnosis and gradually declines thereafter [18,23,24]. The distribution of thrombotic timing observed in this review is generally consistent with these epidemiological findings. With regard to prognosis, sufficient data were not available in the included cases to allow meaningful conclusions. However, cancer-associated thrombosis is known to be associated with increased mortality. Previous studies have reported that VTE is an independent predictor of death within one year after cancer diagnosis (HR 1.6-4.2, P < 0.01) [18,19]. Prognostic data specifically for Trousseau syndrome associated with prostate cancer remain extremely limited, highlighting the need for further case accumulation and clinical studies.

Strengths of the Study

The main strength of this study lies in its systematic synthesis of reported cases of Trousseau syndrome specifically associated with prostate cancer. Although epidemiological studies have examined the association between prostate cancer and thrombosis, most have focused primarily on VTE. In contrast, few studies have specifically addressed Trousseau syndrome as a broader clinical entity that includes both arterial and venous thrombosis [25,26]. This review, therefore, helps address an existing knowledge gap.

Furthermore, this study was conducted in accordance with the PRISMA guidelines and included clearly defined inclusion and exclusion criteria. Clinical data were extracted and organized at the individual case level, allowing detailed evaluation of multiple clinical parameters, including timing of thrombotic onset relative to cancer diagnosis, tumor stage, and PSA level, thrombotic location, diagnostic methods, treatment strategies, and clinical outcomes. By integrating these clinical elements, this review provides a more comprehensive overview of the clinical patterns of prostate cancer-associated Trousseau syndrome.

Importantly, the findings that thrombosis may precede the diagnosis of prostate cancer and that most reported cases occurred in advanced-stage disease highlight the importance of considering underlying malignancy when evaluating unexplained thrombotic events. In addition, the detailed case-level synthesis may serve as a hypothesis-generating foundation for future prospective studies or large database analyses [27]. Potential biological mechanisms in prostate cancer may differ from those classically emphasized in mucin-producing adenocarcinomas. In advanced prostate cancer, mucin-independent pathways, such as TF-bearing extracellular vesicles, platelet-monocyte activation, and systemic coagulation activation, may be more relevant [21,22]. In addition, ADT may contribute indirectly through endothelial and thrombo-inflammatory effects [20,21].

Limitations

This study has several limitations. First, because the analysis was based on case reports, the findings are susceptible to publication bias, and rare or severe presentations may be overrepresented. Second, the small number of cases prevents reliable estimation of incidence or identification of independent risk factors. Third, the level of detail reported in individual case reports varied substantially. In several cases, information on prognosis, follow-up duration, and treatment details was incomplete, limiting comparability across cases. Finally, this study is a descriptive review rather than an observational study; therefore, causal relationships cannot be established. Future research, including prospective cohort studies and large-scale database analyses, will be necessary to clarify the risk stratification of arterial and venous thrombosis in prostate cancer.

## Conclusions

Trousseau syndrome associated with prostate cancer is rare but has been reported primarily in patients with advanced-stage disease and markedly elevated PSA levels. Thrombotic events can occur in both arterial and venous systems and may involve diverse vascular territories. Importantly, thrombosis may precede the diagnosis of prostate cancer in a substantial proportion of patients. Therefore, in cases of unexplained thrombotic events, particularly arterial or multifocal thrombosis in older men, clinicians should consider the possibility of underlying malignancy, including prostate cancer. Evidence regarding the optimal anticoagulation strategy for prostate cancer-associated thrombosis remains limited, particularly for arterial thrombotic events. Future research should include registry-based studies and large-scale database analyses to better define incidence, clinical patterns, and risk factors, as well as mechanistic investigations to clarify the biological pathways underlying thrombosis in prostate cancer. In addition, standardized reporting of cancer-associated thrombosis in case reports and case series would improve comparability across studies and strengthen the evidence base.

## Appendices

Appendix A: Full search strategies

PubMed

("Prostate cancer"[tiab] OR "Prostatic neoplasms"[Mesh] OR "Prostate carcinoma"[tiab]) AND ("Trousseau syndrome"[tiab] OR "Cancer-associated thrombosis"[tiab] OR "Arterial thrombosis"[tiab] OR "Ischemic stroke"[tiab] OR "Hypercoagulability"[tiab])

Filters: English language; publication date from January 1, 2000 to April 30, 2025.

Embase

('prostate cancer':ti,ab OR 'prostatic neoplasm'/exp OR 'prostate carcinoma':ti,ab) AND ('trousseau syndrome':ti,ab OR 'cancer associated thrombosis':ti,ab OR 'arterial thrombosis':ti,ab OR 'ischemic stroke':ti,ab OR 'hypercoagulability':ti,ab)

Limits: English language; publication years 2000-2025.

Web of Science

TS=("Prostate cancer" OR "Prostatic neoplasms" OR "Prostate carcinoma") AND TS=("Trousseau syndrome" OR "Cancer-associated thrombosis" OR "Arterial thrombosis" OR "Ischemic stroke" OR "Hypercoagulability")

Limits: English language; publication years 2000-2025
